# Hepatocellular carcinoma (HCC): An update on risk factors, surveillance, diagnosis and treatment strategies

**DOI:** 10.1016/j.clinme.2025.100532

**Published:** 2025-11-20

**Authors:** Sanju Mathew, Christopher Cussens, Marinos Pericleous

**Affiliations:** a Royal Surrey NHS Foundation Trust, Guildford, Surrey, UK; b University of Surrey, Royal Surrey Foundation NHS Trust, Guildford, Surrey, UK

## Abstract

Primary liver cancer is among the fastest-rising causes of cancer-related death in the UK, and the incidence in the UK has increased by almost 50% over the past decade. With increasing incidence of alcohol and metabolic-related liver disease, the rise in cases is expected to continue. While treatable and potentially curable in early stages, it often presents late, owing to the frequently silent nature of liver disease: it is thought that 50% of patients with HCC are unaware of their diagnosis. In the UK, risk of both developing of liver disease and of death from HCC is higher in areas of social deprivation. The recent publication of the NHS 10-year plan and its emphasis on tackling health inequality make this CME article particularly pertinent. With early detection of liver disease and HCC key for favourable outcomes, it is vital that healthcare professionals are aware of the aetiology and surveillance strategies for HCC to optimise liver disease, and to identify disease for curative treatment.

## Introduction

Primary liver cancer is the sixth most common cancer globally, but the third most common cause of cancer-related death worldwide. Approximately 85% of all primary liver cancers are hepatocellular carcinomas (HCC), with HCC occurring almost exclusively in the context of underlying liver disease.^1^

5-year survival for HCC in England is only 13%, with disease often presenting at an advanced stage with limited treatment options.^2^ Multiple lines of treatment do exist however, through interventional radiology, surgery, transplantation, radiotherapy and systemic therapy. Underlying liver disease and portal hypertension are key considerations in treatment decisions, and may be prohibitive to cancer therapy.

This CME article aims to provide an outline for the common aetiologies, diagnostic criteria, surveillance strategies and treatment considerations for HCC, and how we can strive to improve early identification and treatment outcomes for HCC in the UK.

### Epidemiology and risk factors

**Fig. 1a:**
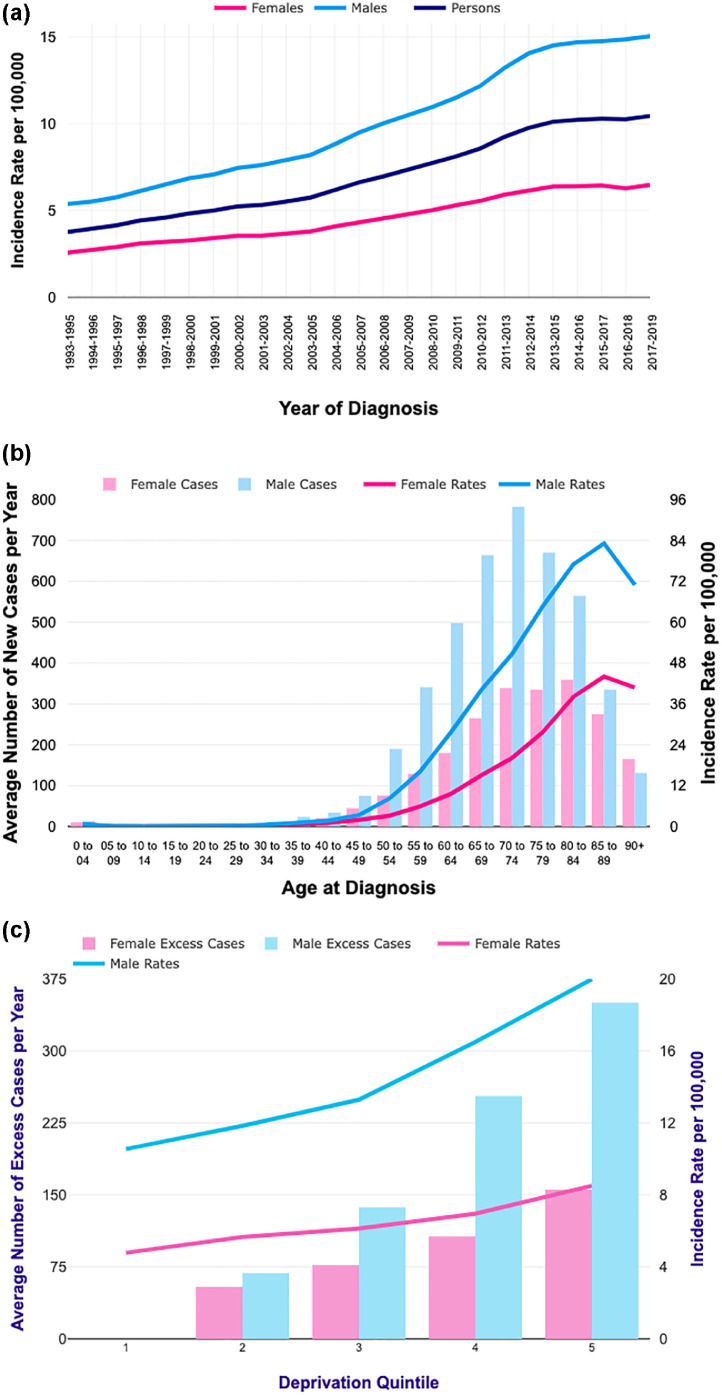
Fig 1a and 1b: Rising incidence of liver cancer in the UK in both men and women, with a peak incidence between 70–74 years for men and 80–84 for women. Figure 1c: Incidence of liver cancer is associated with Index of Multiple Deprivation (IMD) in both men and women. The graph shows the estimated average number of excess cases per year and European age-standardised incidence rates per 100,000 population, by deprivation quintile, England, 2013–2017 Source: Cancer Research UK.^3^

In the UK, HCC is the 17th most common cancer, but the eighth most common cause of cancer-related death.^3^ HCC incidence has increased by almost 50% over the past decade (Fig. 1a and b), alongside a disproportionate rise in the burden of liver disease. More than 90% of patients with HCC have underlying liver disease. Drivers for HCC and liver disease therefore overlap, with cirrhosis being the strongest risk factor.^2^^,^^4^ Older age (with incidence rising steeply from age 40 to 44 years), male sex and deprivation index are independent risks for HCC (Table 1). Globally, chronic viral hepatitis (CVH) infection from hepatitis B (HBV) and hepatitis C (HCV) is the leading driver for HCC, followed by alcohol and metabolic risks. In the UK, alcohol, obesity and metabolic risks are the main causes for liver disease, and the dominant concern therefore in HCC.^2^ Social determinants, health inequalities and deprivation are particular concerns for HCC in the UK, with rates of liver disease and HCC varying across England. Death from liver disease is up to five times more common for those who live in the most deprived areas, and death from HCC is more common in areas of deprivation^2^^,^^3^ (Fig. 1c).

**Table 1:** Aetiology of hepatocellular carcinoma (HCC) and risk factors associated with higher incidence of HCC.

Aetiology of liver disease (HCC)	Risk factors of HCC
Alcohol	Age
Metabolic dysfunction-associated steatotic liver disease (MASLD)	Male sex
Metabolic dysfunction-associated steatotic liver disease (MASLD) patients who drink alcohol (MetALD)	Deprivation
Hepatitis B infection (including non-cirrhotic)	Cirrhosis and underlying liver disease
Hepatitis C infection	
Any cause of cirrhotic including autoimmune liver diseases, hereditary haemochromatosis, α1 antitrypsin deficiency etc.	

Alcohol, MetALD, and chronic viral hepatitis are independently associated with deprivation.

HBV in the UK principally affects patients born and brought up in high-risk countries, but alongside HCV, it is also a risk factor for high-risk groups in injecting and sexually transmitted routes. HBV is classified as a Group 1 carcinogen by the International Agency for Research on Cancer, primarily due to the ability of the HBV virus to integrate directly into hepatocyte DNA. Surveillance in HBV patients is unique in considering younger non-cirrhotic patients who may be at risk of developing HCC from HBV.^5^ But only 50% of patients with HBV are thought to be diagnosed and aware of their disease state, with concerns therefore that patients at risk of HCC development may be unknown and untreated.^6^^,^^7^ In HCV, direct antiviral therapy (DAAs) can achieve cure for HCV in over 95% of patients, but the risk of developing HCC remains in those patients with established cirrhosis (and potentially advanced fibrosis) pre-treatment; and therefore long-term surveillance needs for these patients post-treatment.^5^^,^^8^^,^^19^ MASLD (metabolic dysfunction-associated steatotic liver disease, previously termed NAFLD) is now the leading cause of liver disease, with a (growing) global prevalence estimated at 30%. Age-adjusted mortality for MASLD-related HCC is increasing, and it is important to assess for co-existent aetiologies, particularly the negative effects of alcohol alongside metabolic risks.^2^^,^^9^^,^^10^

### Diagnosis and surveillance

Liver disease and HCC are typically silent, and in the absence of surveillance or incidentally detected lesions, most patients will only be identified in the context of advanced liver disease or advanced HCC.

All cancer care in the UK is managed through dedicated multidisciplinary cancer teams (MDT), with HCC unique in its requirement to consider both the liver (often the dominant risk) as well as the hepatic cancer. The roles of hepatology, hepato-pancreatic biliary (HPB) surgery, interventional radiology and oncology (CNS and consultants) are vital in the MDT. These MDTs are frequently regional, and co-located in HPB surgical centres, or transplant centres across the UK, running a hub-and-spoke model covering the Cancer Alliance regions. Unlike many cancers, HCC diagnosis can be made non-invasively by image criteria (radiology), particularly in early and intermediate stage disease (small volume disease). Cross-sectional imaging with CT and MRI forms the cornerstone of non-invasive diagnosis, with the radiological Li-RADS system developed to given more objective nomenclature to the enhancement characteristics seen in HCC (Table 2).^11^

**Table 2:** LI-RADS® CT/MRI radiological classifications for HCC and LI-RADS® CT/MRI response criteria following treatment.

LI-RADS® v2018 diagnostic categories (imaging criteria limited to those with established liver disease; patients over 18 years, and cannot be used in vascular liver disorders, cardiac congestion, chronic portal vein thrombosis or nodular regenerative hyperplasia^5^)	
LR-NC	If cannot be categorised due to image degradation or omission
LR-1	If definitely benign
LR-2	If probably benign
LR-3	If intermediate probability of malignancy
LR-4	If probably HCC
LR-5	If definitely HCC
LR-M	If probably or definitely malignant but not HCC-specific
LI-RADS® v2018 treatment response	
LR-nonviable	• No lesional enhancement OR • Treatment-specific expected enhancement pattern
LR-equivocal	Enhancement atypical for treatment-specific expected enhancement pattern and not meeting criteria for probably or definitely viable
LR-TR viable	Nodular, mass-like, or thick irregular tissue in or along the treated lesion with any of the following: • Arterial phase hyperenhancement OR • Washout appearance OR • Enhancement similar to pretreatment

The LI-RADS® system and its 2018 update provides radiologists with a classification system that identifies benign lesions (LI-RADS 1), equivocal, indeterminant lesions (LI-RADS 3) that need follow-up, and definitive HCC lesions (LI-RADS 5) that can be treated as HCC, often without the need for a lesional liver biopsy.^11^

It is important to note that this scoring system can only be used in patients with established cirrhosis (and more selectively in high-risk non-cirrhotic HBV), with a pre-test probability that makes a new lesion highly likely to be HCC.^5^ Lesions must be >1 cm, with smaller lesions that cannot be accurately characterised requiring surveillance imaging.^2^ Pathology remains essential in non-cirrhotic lesions, and equivocal lesions where strict radiological criteria are not met. Pathological confirmation is also required in advanced disease where systemic treatment is suitable, unless a decision is made and documented at MDT.^5^

### Biomarkers

The serum glycoprotein alpha-fetoprotein (AFP) is the only validated biomarker currently in HCC, and is typically elevated in advanced HCC, but it may not be secreted in up to 40%, particularly in small HCCs. It may also be elevated in other conditions such as chronic viral hepatitis, cirrhosis, pregnancy, and some other tumours. Sensitivity when used alone for detecting HCC is less than 50%. While other biomarkers and scoring systems exist in clinical studies, none are currently recommended for routine use.

### Surveillance

Surveillance for HCC is widely recommended across international guidelines, with the aim of identifying disease early for curative intent treatment. Surveillance is recommended in patients with cirrhosis, as well as some (often younger) non-cirrhotic patients with hepatitis B.^2^ The clinical trial data to support surveillance, and the groups who benefit most from this intervention, are however not robust. For patients with significant comorbidities, frailty and a performance status at two or above, surveillance is not generally recommended. The same applies for decompensated liver patients, where, in the absence of transplantation, treatment is often not safe to deliver. It remains crucial to involve patients in decision making and rationale for surveillance.^2^

Ultrasound and AFP monitoring 6 monthly is the standard recommended surveillance method, but lesions may be missed with this approach, and ultrasound may give poor views, particularly in obese patients; cross-sectional imaging should be considered for high-risk groups. While surveillance is recommended in MASLD patients with cirrhosis, there is currently no standardised recommendation for surveillance in non-cirrhotic MASLD patients. Prognostic scores, such as the aMAP score,^20^ and specific scores in hepatitis B (PAGE-B)^5^^,^^21^^,^^22^ may help target surveillance to higher-risk individuals, and it remains important to consider individual factors, including co-existing aetiologies towards liver disease.

### Staging and treatment decisions

**Fig. 2:**
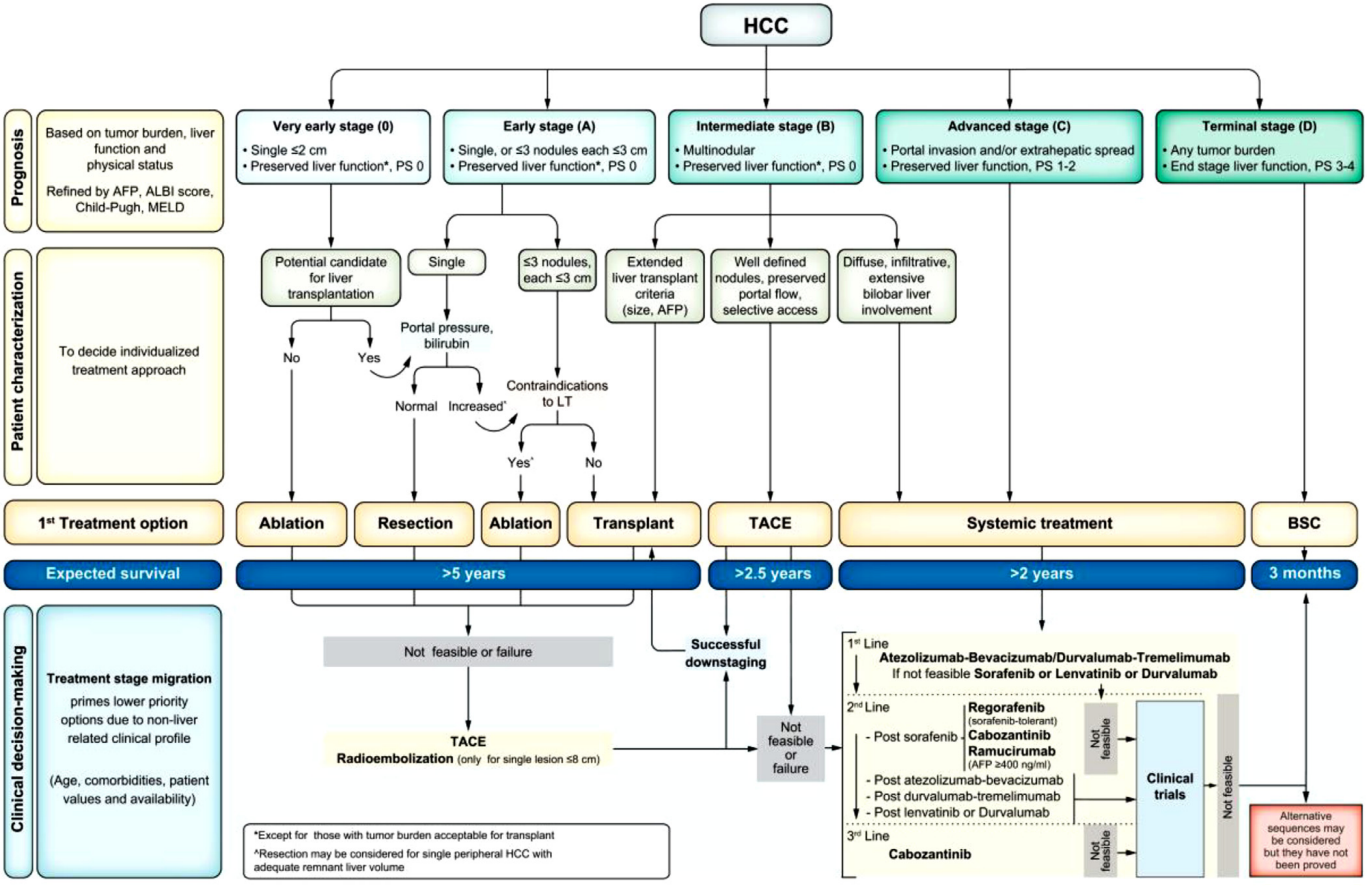
The Barcelona Clinic Liver Cancer (BCLC) system provides staging with regards to the extent of HCC, any extrahepatic disease, and importantly considers hepatic synthetic function and portal hypertension, as well as the Eastern Cooperative Oncology Group (ECOG) Performance Status (PS).^5^^,^^12^ Reprinted from Reig *et al, J Hepatol* 2022, 76: 681–693 ©2022, with permission from Elsevier.

The Barcelona Clinic Liver Cancer (BCLC) system is the most widely used staging system for HCC (Fig. 2).^12^ Unlike most cancer groups that utilise a TNM (tumour, node, metastasis) system, the BCLC staging system is favoured in HCC as it considers liver (synthetic) function, portal hypertensive risks and (cancer-related) performance status variables that can independently impact treatment options and prognosis. Impaired synthetic function or decompensation may leave patients with no available treatment options in the absence of transplantation, despite a low tumour burden.

For very rarly-stage disease, surgical resection or ablation may be used, with 5-year survival rates approaching 80% (with a solitary lesion <2 cm), but recurrence rates of 50–70% at 5 years can be seen post-resection.^5^ Surgical resection and transplant are the mainstay of curative treatment in early-stage disease, with overall 5-year survival more than 60%.^2^ More recently, stereotactic ablative radiotherapy (SABR) has been added as another consideration where standard approaches such as ablation or surgery are not suitable. SABR is normally recommended for tumours less than 5 cm or fewer than six liver lesions in patients who do not have extrahepatic disease or hepatic decompensation.^13^

For intermediate stage disease, the unique dual portal vein (80%) and hepatic artery (20%) supply of the liver allows for locoregional therapy, with transarterial chemoembolisation (TACE) or bland embolisation (TAE) delivered to selectively block off the arterial supply to the cancer, alongside selective internal radiation therapy (SIRT).

Liver transplantation can be considered in early and some intermediate stage disease, and remains the only option for patients with HCC and decompensation. Strict selection criteria is utilised to minimise recurrence (Table 3). More recently, down-staging has been adopted for patients (modestly) outside standard listing criteria at outset, whom may be subsequently eligible for listing if within strict response criteria.^2^^,^^14^

**Table 3:** Transplant criteria for HCC in the UK.

UK HCC criteria for liver transplantation^a^:
• One lesion < 5 cm in diameter • Up to five lesions, maximum diameter 3 cm (all ≤ 3 cm) • Single tumours 5–7 cm with no progression over 6 months • AFP level < 1,000 ng/mL

a Downstaging criteria may be applied for those patients presenting with disease (modestly) outside transplant criteria, and for those patients who exhibit good tumour biology and response to treatment. Extrahepatic disease, macrovascular invasion, tumour rupture and an AFP >1,000 remain contraindications to this consideration.^2^^,^^14^

Systemic therapy is used for patients with compensated cirrhosis not suitable for other interventions, and in more advanced disease. Immunotherapy approaches are now standard of care for first-line systemic treatment, with atezolizumab and bevacizumab (PD-L1 inhibitor and VEGF inhibitor respectively) the most common agents used, and new potential dual-immunotherapy approaches on the horizon. Based on trial data, median overall survival for patients on atezolizumab and bevacizumab was 19.2 months according to the IMbrave150 cohort (NCT03434379).^15^ More recently, we have also seen the approval of the STRIDE regimen (HIMALAYA study – NCT03298451), which employs a dual regimen of a single dose of tremelimumab (anti-CTLA-4 antibody), plus 4-weekly interval durvalumab (anti-PD-L1 antibody). Based on the 5-year survival results, the overall survival rate for patients on tremelimumab and durvalumab was 19.6% months compared to 9.4% on sorafenib.^16^ Patients with advanced disease (BCLC stage D) unfit or unsuitable for treatment, have median survival rates of only 3–4 months, with both cancer and liver disease risks responsible for this poor outlook.^5^

**Fig. 3:**
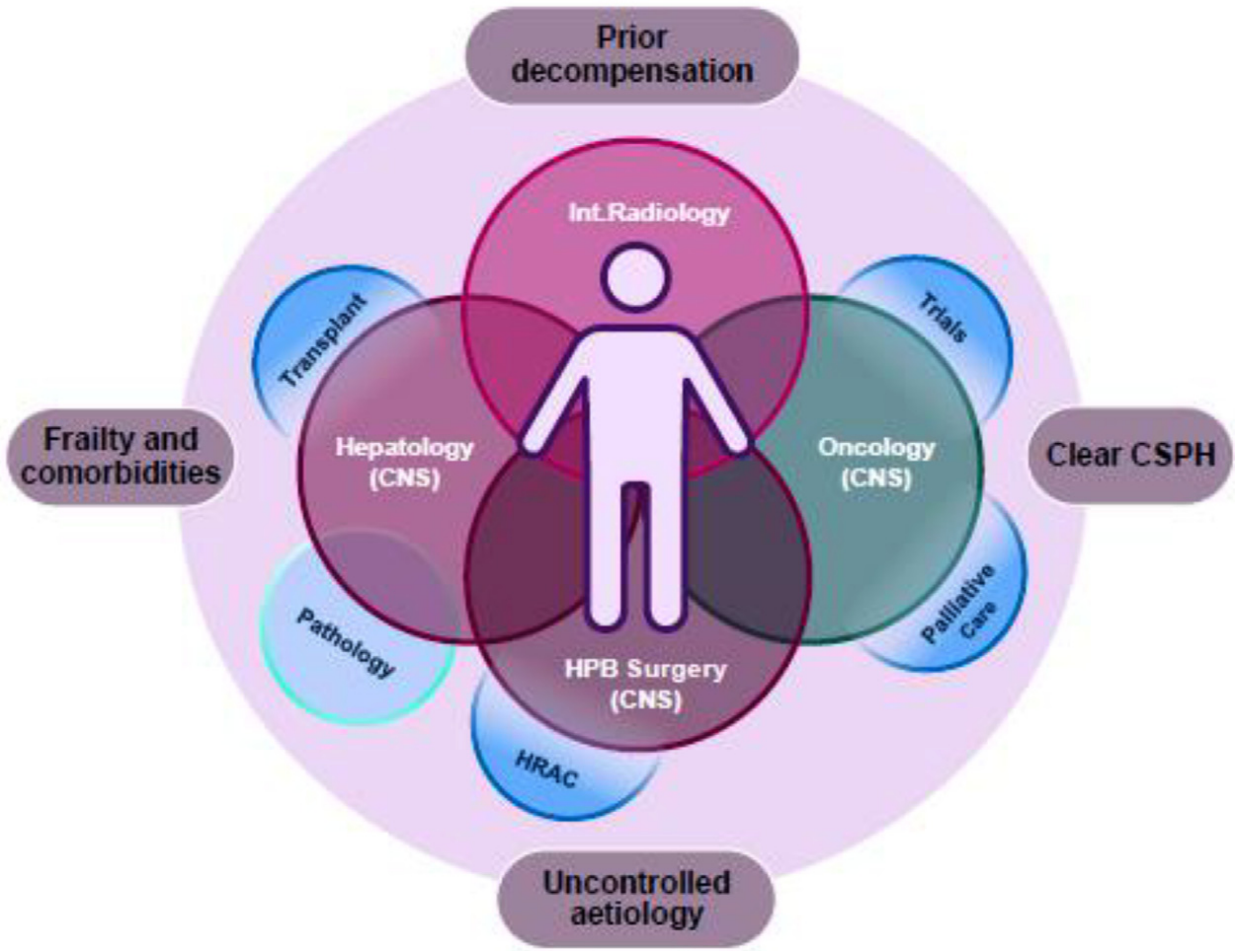
The HCC multidisciplinary team, and considerations in treatment decisions. Int. Radiology, interventional radiology; CNS, clinical nurse specialist; CSPH, clinically significant portal hypertension (patients with low platelet counts, prior decompensation, radiological varices, fibroscan scores >25 kPa may all be suggestive, with invasive measures of hepatic venous pressure gradients (HVPG) that may help decision making for surgical resection).

Decision making for HCC treatment is therefore complex, and an effective HCC MDT requires close working relationships between interventional HPB radiology, HPB surgery, oncology, hepatology and transplant hepatologists. Palliative care support and clinical trials should also be factored in (Fig. 3).

## Discussion

Primary liver cancer is among the fastest-rising causes of cancer-related mortality in the UK, with an incidence to mortality ratio of HCC approaching one.^2^ While the current 5-year survival of HCC in England is less than 20%, early identification can potentially lead to 5-year survival rates of 80% in very early stage disease,^5^ highlighting the importance of surveillance in identifying disease early in higher-risk patients. Cancer Alliance data in England suggest, though, that most patients present outside surveillance systems, via acute or emergency pathways with more advanced disease at outset.^17^ Variability is seen across the country, with deprivation and social determinants having particular importance in liver disease and HCC (Fig. 1c). Surveillance in hard-to-reach population groups may need coordinated action between hospital trusts, primary care and community groups, and direct testing approaches in culturally/geographic appropriate settings (including prison settings).

Multiple modes of treatment exist for HCC, including the unique consideration of transplantation in some patients. Decision making is complex, and frequently regionalised within surgical HPB and transplant centres, with specialist HPB interventional radiology, hepatology, oncology and palliative care services.

Systemic treatment evolution with the use of immunotherapy-based approaches has led to more patients starting on treatment, but also with the need for more complex and ongoing hepatology input, including the parallel risks of liver disease decompensation, and immunotherapy toxicity. To this regard, the aspiration for many HCC units is to run, and maintain joint oncology and hepatology clinics, with physician and CNS keyworker presence throughout the patient journey, including in palliative care settings, where the complications of decompensation and liver disease can be unfamiliar to community palliative care services. Moreover, participation of HCC patients in research activities is strongly encouraged and this can be facilitated via joint HCC and palliative care clinics.^18^

## Funding

This research did not receive any specific grant from funding agencies in the public, commercial, or not-for-profit sectors.

## CRediT authorship contribution statement

**Sanju Mathew:** Writing – review & editing, Writing – original draft, Supervision, Methodology. **Christopher Cussens:** Writing – review & editing. **Marinos Pericleous:** Writing – review & editing, Writing – original draft, Supervision, Methodology.

## Declaration of competing interest

The authors declare that they have no known competing financial interests or personal relationships that could have appeared to influence the work reported in this paper.
